# Supplementary material for: MetaQTL: a package of new computational methods for the meta-analysis of QTL mapping experiments
Source: BMC Bioinformatics. 2007 Feb 8;8:49. doi: 10.1186/1471-2105-8-49 (PMC1808479; doi:10.1186/1471-2105-8-49)
Supplement: Additional File 7 — MetaQTL Package : jar file and tutorial. This Zip archive contains both the MetaQTL JAR file and the files of the tutorial. [file 1471-2105-8-49-S7.zip › org.inra.metaqtl/doc/org/metaqtl/main/package-use.html]

Uses of Package org.metaqtl.main


|  |  |  |
| --- | --- | --- |
| **Overview** \| **Package** \| Class \| **Use** \| **Tree** \| **Deprecated** \| **Index** \| **Help** | |  |
| PREV   NEXT | **FRAMES**    **NO FRAMES**     **All Classes** |  |


---


## **Uses of Package org.metaqtl.main**

| Packages that use org.metaqtl.main | |
| --- | --- |
| **org.metaqtl.main** |  |

Classes in org.metaqtl.main used by org.metaqtl.main

****CmdLineParser****
  
          Largely GNU-compatible command-line options parser.

****CmdLineParser.IllegalOptionValueException****
  
          Thrown when an illegal or missing value is given by the user for
an option that takes a value.

****CmdLineParser.Option****
  
          Representation of a command-line option

****CmdLineParser.OptionException****
  
          Base class for exceptions that may be thrown when options are parsed

****CmdLineParser.UnknownOptionException****
  
          Thrown when the parsed command-line contains an option that is not
recognised.

****MetaMain****

---


|  |  |  |
| --- | --- | --- |
| **Overview** \| **Package** \| Class \| **Use** \| **Tree** \| **Deprecated** \| **Index** \| **Help** | |  |
| PREV   NEXT | **FRAMES**    **NO FRAMES**     **All Classes** |  |


---
